# Essential roles of the nucleolus during early embryonic development: a regulatory hub for chromatin organization

**DOI:** 10.1098/rsob.230358

**Published:** 2024-05-01

**Authors:** Bo Fu, Hong Ma, Di Liu

**Affiliations:** ^1^ Institute of Animal Husbandry, HeiLongJiang Academy of Agricultural Sciences, Harbin 150086, People's Republic of China; ^2^ Key Laboratory of Combining Farming and Animal Husbandry, Ministry of Agriculture and Rural Affairs, Harbin 150086, People's Republic of China

**Keywords:** preimplantation embryo, nucleolus precursor bodies, chromatin organization, Dux, totipotency, somatic cell nuclear transfer

## Abstract

The nucleolus is the most prominent liquid droplet-like membrane-less organelle in mammalian cells. Unlike the nucleolus in terminally differentiated somatic cells, those in totipotent cells, such as murine zygotes or two-cell embryos, have a unique nucleolar structure known as nucleolus precursor bodies (NPBs). Previously, it was widely accepted that NPBs in zygotes are simply passive repositories of materials that will be gradually used to construct a fully functional nucleolus after zygotic genome activation (ZGA). However, recent research studies have challenged this simplistic view and demonstrated that functions of the NPBs go beyond ribosome biogenesis. In this review, we provide a snapshot of the functions of NPBs in zygotes and early two-cell embryos in mice. We propose that these membrane-less organelles function as a regulatory hub for chromatin organization. On the one hand, NPBs provide the structural platform for centric and pericentric chromatin remodelling. On the other hand, the dynamic changes in nucleolar structure control the release of the pioneer factors (i.e. double homeobox (Dux)). It appears that during transition from totipotency to pluripotency, decline of totipotency and initiation of fully functional nucleolus formation are not independent events but are interconnected. Consequently, it is reasonable to hypothesize that dissecting more unknown functions of NPBs may shed more light on the enigmas of early embryonic development and may ultimately provide novel approaches to improve reprogramming efficiency.

## 1. Introduction

The nucleolus was first documented in 1835 [1]. Subsequent studies confirmed its intricate association with specific chromosomal loci (i.e. nucleolar organizer regions) and its pivotal role in the synthesis of ribosomal RNA (rRNA) and the assembly of ribosomes [2]. Briefly, fibrillar centres (FCs), the dense fibrillar component (DFC) and the granular component (GC) in the nucleolus, corresponding to different steps of ribosome biogenesis, fulfil rRNA transcription, rRNA processing and assembly of ribosomal subunits and then maintain normal cell growth. This is the typical situation found in somatic cells, stem cells, growing oocytes and early embryos that have completed zygotic genome activation (ZGA).

The nucleolus in fully grown oocytes or early embryos differs greatly from the nucleolus in somatic cells [3]. Unlike the canonical tripartite nucleolus in somatic cells, the nucleolus in fully grown oocytes or early embryos is atypical in appearance, with dense homogeneous fibrillar material rather than the three basic sub-compartments in the typical nucleolus [4]. Atypical nucleoli in fully grown oocytes and early embryos are termed ‘nucleolus-like bodies (NLBs)’ and ‘nucleolus precursor bodies (NPBs)’, respectively. The traditional view held that NLB/NPB only served as a passive repository site of nucleolar proteins and materials, which were gradually used by embryos to assemble fully functional nucleoli when ZGA occurs and ribosome biogenesis resumes [4]. However, somatic cell nuclear transfer (SCNT) experiments revealed that once the nucleolus of fully grown oocytes was removed, the fully functional nucleolus derived from donor somatic cells could not compensate for loss of NLB/NPB materials in reconstructed embryos and the reconstructed embryos arrested, which means that NLB/NPB in oocytes/early embryos may perform different functions and the previous view is untenable [5]. Furthermore, a series of nucleolar transplantation experiments proved that NPBs play essential roles only shortly after fertilization (i.e. 8–10 h post-fertilization) in mouse embryos [5–7]. Given that the 8–10 h post-fertilization is the critical period of development before ZGA, it is interesting to dissect the real functions that NPBs may exert during this specific time window. Current experimental evidence has demonstrated that NPBs provide a regulatory hub for chromatin organization in early embryos. More explicitly, NPBs provide the structural platform for centric and pericentric chromatin remodelling, then maintain the stability of centromeres and ensure correct chromosome segregation [8]. At the same time, NPBs provide a permissive microenvironment for the release of pioneer factors such as double homeobox (Dux) [9,10], which facilitates the establishment of totipotency. Mechanistically, Dux, which resides at the top of the totipotency regulatory hierarchy [11], regulates murine endogenous retrovirus-leucine (MERVL) and two-cell-specific transcripts, then participating in establishing totipotency indirectly [12–15].

Currently, little is known about the mechanisms underlying cell fate transition and ZGA in early embryos. Developmental block and abnormal ZGA remain formidable barriers for *in vitro* embryo production (IVEP). In particular, ZGA is incomplete, and two-cell-specific genes are not properly activated in cloned embryos [16–19]. Concomitant with these defects, incomplete NPB architecture remodelling also exists in cloned embryos [20,21]. We propose that NPBs may be associated with the establishment of totipotency and ZGA by organizing chromatin around the nucleolar periphery. Detailed dissection of the functions of NPBs promises to elucidate the molecular mechanisms governing early development and provide a novel perspective that may pave the way towards reprogramming differentiated somatic cells into totipotent cells.

## 2. Liquid droplet-like properties of nucleolus

**Figure 1.**
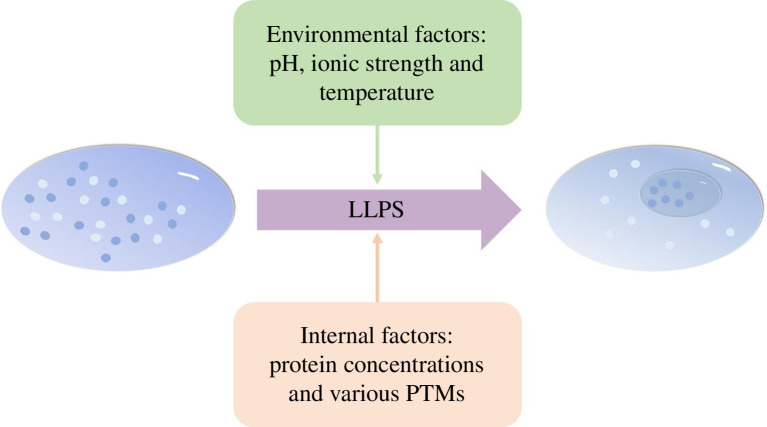
Scheme of liquid–liquid phase separation (LLPS). Proteins carrying multiple modular interaction domains (MIDs) and low complexity domains (LCDs) are involved in LLPS. Environmental factors (such as pH, ionic strength and temperature) and internal factors (including protein concentrations and various PTMs of proteins) affect LLPS by regulating different types of multivalent interactions.

As early as 2011, Brangwynne *et al*. found that nucleoli behave as liquid-like droplets, a feature that determines the size and shape of the nucleolus [22]. The nucleolus, like other membrane-less organelles (such as Cajal bodies, germline P granules, histone locus bodies and nuclear speckles), is formed by liquid–liquid phase separation (LLPS) of DNA, RNA and protein mixtures [23]. More colloquially, solute and solvent molecules are evenly distributed in solution; once LLPS occurs, the solute molecules condense to form a membrane-less liquid-like concentrated phase, then leave surrounding solvent molecules to form a dilute phase, resembling oil and water de-mixing [24]. These two separated phases can also merge into one phase, and the transition between phases is determined by environmental factors (such as pH, ionic strength and temperature), protein concentrations and various post-translational modifications (PTMs) of proteins (including phosphorylation, acetylation, methylation and deamidation). When the physiological factors reach the transition point, liquid droplets are formed, with multivalent protein–protein and nucleic acid–protein interactions acting as the driving force [25–29]. At this time, dense coacervate liquid droplets separate from the dilute phase through LLPS, also known as condensation or coacervation [30]; thus, the resulting nucleolus is denser than the surrounding nucleoplasm (figure 1).

Notably, some special non-coding RNA, such as aluRNA, also participates in liquid droplet formation of the nucleolus. In brief, aluRNA (RNA Pol II transcripts derived from intronic Alu elements) can recruit nucleolin and nucleophosmin to the nucleolus. Overexpression of aluRNA increases nucleolar size and upregulates pre-rRNA expression, thus affecting nucleolar structure and function [31].

Because the nucleolus exists in the form of liquid-like droplets, the question arises as to whether the content of a nucleolus mixes and fuses into a single liquid phase. Indeed, the multi-layered compartments (i.e. FC, DFC and GC) in a nucleolus represent distinct, coexisting liquid phases [32]. In essence, differences in the biophysical properties of the phases cause layered droplet organization, with droplet surface tension (derived from amino acid sequence-dependent properties of molecular components) playing a prominent role. Schematically, differences in intrinsically disordered proteins or regions result in differences in the miscibility of proteins contained in different nucleolar compartments, providing individual compartments with different solvent properties and keeping different compartments phase-separated to ultimately form the canonical tripartite nucleolus [32].

Liquid phase immiscibility and sub-compartmentalization in the nucleolus may have important functional implications. Phase separation and coexistence of multiple liquid RNA/protein phases may facilitate spatial localization and processing of molecules in a membrane-less environment. Because of its liquid droplet-like properties, the nucleolus is also endowed with unique structural and biological features. For instance, functions of the nucleolus can be rapidly switched on and off by controlling the formation and dissolution of the liquid droplet, and the liquid droplet-like structure makes it possible to rapidly exchange biomolecules between nucleoli and the nucleoplasm [26,33]. In addition, the nucleolus can elevate reaction rates by concentrating rRNA processing factors [32]. More importantly, dissecting the functions of NPBs in zygotes or early embryos has involved a series of micromanipulations targeting the NPBs, and recent papers have already given thorough descriptions for this [7,34]; it is the liquid-like biophysical properties of NPBs that makes the nucleolus amenable to various micromanipulation procedures. For instance, NLBs isolated from fully grown mouse oocytes can come into close contact with each other and then fuse [35]. Moreover, NLBs in germinal vesicles (GVs) or NPBs in pronuclei can penetrate the nuclear envelope [34], which also exhibits liquid droplet-like properties. We should bear in mind that causing no irreparable damage to chromosomes is the first prerequisite for nucleolar transplantation approaches. Given the tight association between NPBs and chromatin, one concern was the possibility of DNA damage during enucleolation. However, levels of phosphorylated H2A.X, a marker of DNA damage, showed no significant increase after enucleolation by micromanipulation, and then removal of NLBs caused no DNA damage [8], which was due to the liquid droplet-like properties of NLBs. The reason for this may be that the nucleolus is sequestered in the nucleus by liquid droplet formation through LLPS [36], so NLBs/NPBs behave like a liquid-like droplet [22]. When NLBs or NPBs enter the manipulation pipette, the penetrated nuclear envelope seems to work as a filter, retaining all the chromatin inside the nuclear envelope without chromatin loss.

## 3. The composition and formation of nucleolus in oocytes and early embryos

In contrast to the nucleolus in somatic cells, the nucleolus (NLB/NPB) in fully grown oocytes or early embryos exhibits entirely compact and homogeneous spherical bodies whose mass is composed of packed fibrous materials [4,37]. A series of nucleolar transplantation experiments demonstrated that enucleolated oocytes are unable to rebuild NPBs in zygotes, and NPBs derived from two-cell stage embryos can rescue the developmental competence of enucleolated oocytes [5,38], which suggests that NLBs in oocytes and NPBs in zygotes are similar in function. To date, the vast majority of studies have been conducted on oocytes to analyse nucleolar components; therefore, to be more precise in academic descriptions, currently we cannot unequivocally state that NLBs and NPBs are similar in their components. The protein content of NLB varies by species. For example, a single mouse NLB contains approximately 1.55 ng of total protein, while porcine NLB contains 0.90 ng [39]. A model of interspecies NLB transplantation between mouse and pig oocytes has also shown that the dosage of key nucleolar factors, rather than the species origin, affects embryonic development [39]. Previous studies have shown that NLBs do not contain lipids, polysaccharides or DNA [40–42]. Generally, assembly of membrane-less organelles depends on scaffolding proteins, which have intrinsically disordered regions or multivalent domain arrays, and RNA facilitates the overall assembly process [26,43]; therefore, it is natural to think that proteins and RNA play key roles in the assembly of the nucleolus in oocytes or early embryos. Recently, Shishova *et al*. optimized conditions for staining paraformaldehyde-fixed oocytes with acridine orange and fluorescein-5-isothiocyanate to detect RNA and proteins in oocytes, then demonstrated that proteins and RNA are major components of NLBs. Shishova *et al*. further found that B23 (or nucleophosmin), C23 (or nucleolin), fibrillarin and upstream binding factor (Ubtf) were immersed in the NLB mass, and NLBs lack rRNA [44]. Furthermore, Ogushi *et al*. isolated nucleoli from fully grown oocytes through a nucleolar transplantation approach, and then determined the protein composition by mass spectrometry (MS) analyses. Bearing in mind the technical defects of the nucleolar transplantation approach, isolated nucleoli inevitably include some components derived from the nucleoplasm and cytoplasm. Thus, the localization of candidate proteins, which were identified by MS analysis, should be evaluated by expressing N-terminal enhanced green fluorescent protein (eGFP)-tagged fusion proteins of candidate proteins. Eventually, NPM2, NCL, SSRP1 and NOLC1 were confirmed to be localized at the nucleolus [45]. Among these nucleolar components, the function of NPM2 is particularly noteworthy. NPM2 is required for the maintenance of nucleolar structure and chromatin compaction in oocytes. In detail, as in NPM2−/− oocytes, nucleolus structure was not observed in NPM2-null one-cell embryos; meanwhile, hypoacetylated histone H3 was undetectable in NPM2-null one-cell embryos, which means that NPM2 may play a key role in heterochromatin formation that surrounds the nucleolus in oocytes and early zygotes [46]. The K-rich motif in the C-terminus of NPM2 is essential for the targeting of NPM2 to the nucleolus. From a mechanistic perspective, the K-rich motif contains several lysine residue pairs (positions 192/193, 201/202 and 206/207) and a single lysine residue at position 195, thus the lysine residues might act cooperatively to regulate targeting of NPM2 to the nucleolus [47]. Ogushi *et al.* also found that apart from the C-terminus of NPM2, which contains the K-rich motif, the N-terminal core domain of NPM2 is also responsible for oocyte nucleolus assembly. The truncation mutants of NPM2, which lack a core domain, only concentrated in the nucleoplasm and were excluded from the nucleolus [45].

Early embryonic development is accompanied by dramatic structural rearrangements of the nucleolus. When the oocytes are still in the growth phase, RNA Pol I is active, and the nucleolus can still be divided into the three basic sub-compartments, that is, FCs, DFCs and GCs, which indicates that the nucleoli in growing oocytes resemble fully functional nucleoli in differentiated somatic cells [48,49]. As oocytes grow, RNA Pol I activity and rRNA synthesis is gradually shut down. Once oocyte growth is complete, RNA Pol I activity disappears and the nucleolus in fully grown oocytes is transformed into a compact mass (NLB) composed only of dense fibrillar materials [4]. It is well established that the fully functional nucleolus in somatic cells is dissolved in the cytoplasm at the beginning of every cell division, and when nuclei reform, nucleolar material reappears in the newly formed nuclei. The NLBs, like fully functional nucleoli in somatic cells, also disperse in the cytoplasm coinciding with GV breakdown and the onset of meiotic maturation. After fertilization and male/female pronucleus formation, NPBs, structures like the NLBs, reappear in pronuclei. NPBs received their name because NPBs provide the building blocks for fully functional nucleoli during re-initiation of RNA Pol I transcription, and fully functional nucleoli are always associated with NPBs, either on their surface or inside, during the production of new ribosomes in embryos [37,50]. Following ZGA, a typical nucleolus is formed at the morula stage and the original NPBs disappear [50]. On the contrary, enucleolation of oocytes prior to activation showed that nucleolar materials cannot be resynthesized in fertilized zygotes [5]. Based on the above phenomena, it would appear that NLBs/NPBs provide the reserve substances for embryos to form fully functional nucleoli when ribosome biogenesis restarts. However, recent results do not lend support to this view. In experiments performed by Ogushi *et al*., NLBs were microsurgically removed from fully grown oocytes before GV breakdown; after enucleolation and maturation of oocytes, the nucleus of cumulus cells or embryonic stem cells (ESCs) was injected into the cytoplasts of enucleated oocytes at metaphase II, then the reconstructed oocyte was activated artificially. Unexpectedly, no nucleolus was seen in newly formed pseudo-pronuclei in these reconstructed embryos, which ultimately arrested after a few cleavages [5]. Logically, if the function of NLBs/NPBs was only to provide the reserve substances for rebuilding fully functional nucleoli in embryos, then fully functional nucleoli in donor cells should compensate for loss of NLB/NPB materials in reconstructed embryos. However, fully functional nucleoli in somatic/ESC nuclei could not substitute for the original NLB/NPB materials in reconstructed embryos, and these early embryos were arrested, which indicated that the components of NLBs/NPBs in oocytes/early embryos may be very distinct from that of fully functional nucleoli and may perform different functions. Furthermore, a series of nucleolar transplantation experiments proved that NPBs were indispensable for early embryonic development, rather than providing support for fully functional nucleolus re-establishment. In particular, the time window when NPBs play essential roles was narrowed down to approximately 8–10 h post-fertilization in mouse embryos [5–7]. That is, once early embryos pass a critical window of development, enucleolation may not damage early embryos, and these modified embryos can develop further and assemble fully functional nucleoli in the absence of NPB materials after ZGA. However, key questions remain, including what function the nucleolus exerts during 8–10 h post-fertilization and how these functions are orchestrated.

## 4. Nucleolus precursor bodies involved in centric and pericentric chromatin remodelling

**Figure 2.**
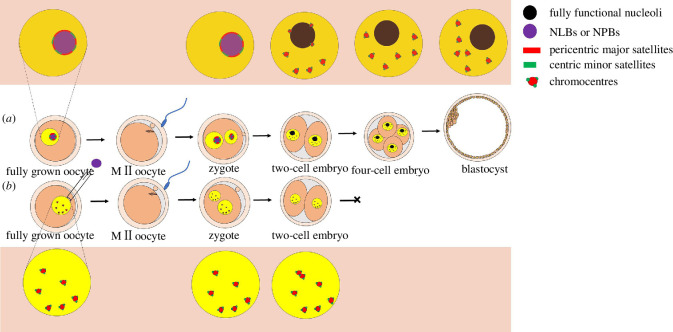
Scheme of the nucleolar cycle and localization of centromeres during mouse early embryonic development. (*a*) Nucleolar cycle and localization of centromeres in normal embryos derived from nucleolus-intact oocytes. NLBs, the nucleolus in fully grown oocytes, are composed only of dense fibrillar materials. Centric and pericentromeric regions are localized around NLBs. As oocytes mature, NLBs are dissolved in the cytoplasm. After fertilization, the nucleolus structure reappears in zygotes in the form of NPBs, and pericentromeric heterochromatin maintains a close association with the periphery of NPBs to form a ring-like structure. At the late two-cell/early four-cell stage transition, centric and pericentric regions detach from the periphery of NPBs and begin to regroup into structures that resemble classical chromocentres, with NPBs replaced by a fully functional nucleolus. Ultimately, these embryos can develop to the blastocyst stage. (*b*) Nucleolar cycle and localization of centromeres in abnormal embryos derived from nucleolus-null oocytes. Although nucleolus-null oocytes can still undergo maturation and fertilization, no NPBs were observed in the pronuclei of resulting zygotes. After NLBs in the fully grown oocytes were removed, centromeres were equally scattered throughout pronuclei in NPB-null zygotes, with several signals of centromeres forming clusters morphologically resembling chromocentres. Ultimately, NPB-null embryos are arrested at the two-cell stage.

The specific morphological configuration of heterochromatin is characteristic of zygotes. It is well known that the nucleolus, together with the nuclear lamina, serves as a compartment for the location and regulation of inactive heterochromatin [51]. Generally, a very small amount of heterochromatin appears in zygotes or early embryos, with the pericentric region accounting for most of the heterochromatin. Centric regions are organized around minor satellites, which are flanked by pericentric regions composed of A/T-rich major satellites. In differentiated somatic cells, pericentric and centric regions from different chromosomes cluster together, and then form chromocentres that can be visualized as bright foci with 4',6-diamidino-2-phenylindole (DAPI) [52]. The minor satellite repeats comprise the structural framework for kinetochore and the major satellite repeats form the pericentric region for chromosome cohesion [53,54]; then the chromocentre contributes to chromatin organization and chromosome segregation. Unlike in somatic cells, the chromocentre is not present in zygotes or early embryos before ZGA, while the centric and pericentromeric regions are located on the periphery of NPBs, forming a pericentromeric heterochromatin ring [55]. In more detail, although DAPI-dense chromocentres can still be observed in growing oocytes that contain a fully functional nucleolus; after fertilization, nucleolus material reappears in female/male pronuclei in the form of NPBs, and the chromocentres disappear. At this time, NPBs act as a structural platform for centric and pericentric chromatin location, and this spatial arrangement of centromere regions at the NPB periphery is a common feature at the early stage of development in mammals [55–58]. During ZGA, chromocentres gradually form at the late two-cell stage of mouse embryos and centric and pericentric regions begin to regroup into chromocentres [20,59,60]. Concomitantly, NPBs are replaced with a fully functional nucleolus (figure 2).

Considering the dynamic reorganization of pericentric regions during early embryonic development, it is natural to raise the question: what are the implications of anchoring pericentric regions to the NPB periphery? Fertilization is followed by genome-wide epigenetic reprogramming, which includes de novo establishment of chromatin domains such as the formation of the pericentric heterochromatin. Acquisition of this highly compact chromatin organization in pericentric regions ensures the subsequent kinetochore loading and progression through the first mitosis. As revealed by recent studies, after pronucleus formation, centromeres are localized around NPBs in normal zygotes, while centromeres are equally distributed throughout pronuclei in NPB-null zygotes, with several centromeres forming clusters morphologically resembling chromocentres [6,44]. Jachowicz *et al*. proposed that the peripheral localization of NPBs at pericentromeric regions is a prerequisite for heterochromatic silencing because this spatial configuration of pericentromeric regions always occurs before the appearance of the heterochromatin signatures such as mono- and tri-methylation of histone 3 on lysine 27 (H3K27me1 and H3K27me3) and heterochromatin protein 1β [61]. Fulka & Langerova demonstrated that NPBs provided a structural platform for centric and pericentric chromatin remodelling [8]. Death domain-associated protein 6 (DAXX), a H3.3 histone chaperone, plays an essential role in chromatin silencing, particularly in the pericentromeric areas [62]. After the removal of NPBs, DAXX, which is localized to pericentric heterochromatin regions in normal zygotes, cannot bind to pericentric regions in the nucleolus-less zygote, resulting in a significant reduction in major and minor satellite DNA by 12% and 18%, respectively. Furthermore, Fulka & Langerova found that removing NPB causes extensive chromosome bridging during the first embryonic mitosis, which may cause aberrant mitosis and developmental arrest [8]. Studies by Ogushi *et al*. also confirmed that abnormal heterochromatin formation around pericentromeric regions caused chromosome missegregation, as well as mitotic delay in nucleolus-less zygotes [45]. Taken together, it is NPBs rather than chromocentres in zygotes that provide structural support for centric and pericentric chromatin remodelling, thus ensuring correct chromosome segregation and proper embryonic development.

## 5. Nucleolus precursor bodies correspond to potency and plasticity

As described above, in the early mouse embryo, the maintenance of NPBs and the remodelling of pericentromeric heterochromatin occur prior to the late two-cell stage. Coincidently, major ZGA also occurs during the late two-cell/early four-cell stage transition, accompanied by the transition from totipotency to pluripotency. Moreover, reduced rRNA output and immature nucleolus structures, which resemble NPBs in two-cell-stage embryos, were also found in two-cell-like cells that resemble two-cell-stage blastomeres and exhibit totipotency-like developmental potential [12]. In particular, disturbing fully functional nucleolus structures of embryonic stem cells via blocking RNA polymerase I activity or preventing nucleolar phase separation can also facilitate the conversion from the pluripotent state to the totipotent state [9,10]. All these phenomena indicate that NPBs in early embryos correspond to totipotency. Therefore, the linkage between NPBs and totipotency inspired further study.

The embryonic transcription factor Dux regulates chromatin opening, MERVL and two-cell-specific transcripts in totipotent cleavage-stage mouse embryos, so is involved in the establishment of totipotency and the ZGA process. Three *Dux* genes (*DUXA, B* and *C*) encode proteins with an N-terminal double homeodomain, but only the *DUXC* branch harbours a conserved C-terminal activation domain, which permits *DUX4* in humans or *Dux* in mice to induce the expression of their target genes [63]. For example, *Dux* is targeted to MERVL and two-cell-specific genes by its two N-terminal homeodomains and recruits the histone acetyltransferase p300–cAMP response element-binding protein (CREB) complex to local targets via its C-terminal domain, then opens chromatin around the transcription start sites of target genes [13–15,64,65]. Recent results also identified five approximately 100 amino acid repeats followed by a single 14 amino acid highly acidic tail in the C-terminus of *Dux* and further demonstrated the cooperativity between active repeats and the acidic tail, probably facilitating cofactor recruitment, *Dux*-mediated opening of targets and transcription [66].

Since 2012, MERVL transcripts have been used as a marker of totipotency [12]. In principle, ZGA is characterized by the massive reactivation of endogenous retroviruses, which provide long terminal repeats as stage-specific cis-regulatory elements (e.g. alternative promoters) to drive a subset of two-cell genes and generate chimeric transcripts with the host genes [67]. Thus, fulfilment of ZGA results in totipotent blastomeres that are equipped with the potential to produce both embryos and extraembryonic appendages [68]. Recent results further revealed that full-length MERVL transcripts are indispensable for accurate regulation of the host transcriptome and chromatin state during preimplantation development, therefore, repressing MERVL may result in embryonic lethality [69].

Given all the above facts, it is believed that *Dux*, as an activator of MERVL, is one of the key drivers of totipotency and ZGA [11].

Recent studies demonstrated that direct physical interaction with nucleolar components regulates *Dux* activation, indicating that NPBs are associated with totipotency. *DUXC*-family homologues, such as *DUXC*, *DUX4* and *Dux*, show a macrosatellite tandem-array organization. For instance, the human D4Z4 macrosatellite has 11–150 3.3 kb repeats, with each repeat nested with a copy of an intron-less *DUX4* [70]. Németh *et al*. also found that D4Z4 macrosatellite repeats exhibit the feature of nucleolus-associated chromatin domains (NADs), where heterochromatin is spatially concentrated and enriched with repressive histone markers (such as H3K9me3, H3K27me3 and H4K20me3), indicating that NADs may provide a suppressive microenvironment for *Dux* expression [71,72]. Indeed, in differentiated somatic cells, *DUX4* and *DUX4* target genes are generally repressed and once the epigenetic repression of the *DUX4* loci in somatic tissues becomes less efficient, misexpression of *DUX4* can cause muscular dystrophies, such as facioscapulohumeral dystrophy (FSHD) [73]. However, after fertilization, *Dux* genes in zygotes or early two-cell embryos are transcribed during minor ZGA, then activate downstream target genes (including MERVL and two-cell-specific genes) and facilitate the establishment of totipotency and ZGA [13–15]. Embryo DNA fluorescence *in situ* hybridization (FISH) carried out with *Du*x oligo probes also revealed that *Dux* loci are located in the nucleoplasm but not at the NPB periphery, which is closely linked to *Dux* expression [9]. This indicated that *Dux* loci were released and NPBs provided the permissive microenvironment for *Dux* expression. As fertilized embryos develop to the late two-cell stage, when embryos undergo transition from totipotency to pluripotency and NPBs are replaced with a fully functional nucleolus, long interspersed nuclear element 1 RNA (LINE 1 RNA) serves as a nuclear RNA scaffold to recruit nucleolin (the component of matured nucleoli) and Kruppel-associated box-associated protein 1 (Kap1) to *Dux* loci [74]. Since nucleolin, together with Kap1, forms the nucleolin/Kap1/LINE 1 complex that mediates *Dux* repression and rRNA expression, then anchoring *Dux* loci to the nucleolus periphery can repress *Dux* gene [74]. The DNA FISH experiment also confirmed that *Dux* loci relocate from the nucleoplasm to the nucleolar periphery during the transition from totipotency to pluripotency, which is tightly associated with *Dux* repression [9]. Recent studies revealed that in the process of nucleolin/Kap1 complex inhibiting *Dux* gene, LIN28 played its corresponding role. In detail, LIN28A coordinates with nucleolin, fibrillarin and nucleolar RNAs to promote rRNA biogenesis, then contributing to the nucleolar integrity. The more important thing is that LIN28A resides in the nucleolin/Kap1 complex, with this complex organized around the nucleolin/Kap1 complex, with this complex organized around the *Dux* loci at the peri-nucleolar region. Namely, LIN28A mediates nucleolin/Kap1 occupancy on the *Dux* loci to repress *Dux* expression. Once LIN28A is knocked out, the LIN28-mediated complex was interrupted, then *Dux* repression terminated, which indicates that *Dux* regulation by the nucleolin/Kap1 complex is LIN28-dependent [75].

**Figure 3.**
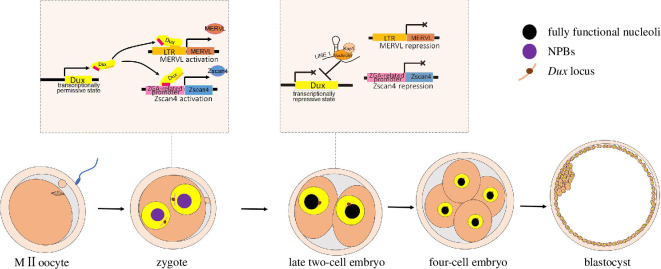
Scheme of dynamic interactions between *Dux* loci and the nucleolus, as well as *Dux* activation. After fertilization and male/female pronucleus formation, NPBs appear in the pronuclei and *Dux* loci are located in the nucleoplasm, providing the permissive microenvironment for *Dux* expression. Then, *Dux* drives the expression of MERVL and ZGA-related genes, such as the zinc finger and SCAN domain containing 4 gene (*Zscan4*) via binding to a long terminal repeat (LTR) or ZGA-related promoter, ultimately establishing totipotency. As the fertilized embryos develop to late two-cell stage, NPBs are replaced with a fully functional nucleolus and LINE 1 RNA recruits nucleolin/Kap1 to *Dux* loci, thus relocating *Dux* loci to the nucleolar periphery. This physical interaction between *Dux* loci and the nucleolus represses *Dux* expression. Without *Dux* activation, neither MERVL nor *Zscan4* is transcribed during the transition from totipotency to pluripotency and embryos exit the two-cell state.

Taken together, in early embryos, the ZGA process is accompanied by structural and functional rearrangements of the nucleolus. The existence of NPBs creates a time window for releasing a pioneer factor (i.e. *Dux*), ultimately participating in totipotency establishment. Loss of physical interaction between the *Dux* loci and NPBs provides the microenvironment for *Dux* expression, which facilitates the establishment of totipotency. Conversely, the tight association between *Dux* loci and the matured nucleolus represses the *Dux* gene, favouring exiting from the two-cell state and conversion from totipotency to pluripotency. The overall procedure is described in figure 3.

Although the above phenomenon showed that *Dux* resides at the top of the totipotency regulatory hierarchy, we should also bear in mind that the acquisition of totipotency includes redundancy. For example, in *Dux* knockout mouse embryos, a minority of *Dux* target genes were affected and the knockout embryos did not arrest at the two-cell stage and survived to adulthood with reduced developmental potential [64]. The primary reason for this survival is that, on the premise of losing *Dux*, ZGA can be redundantly secured by another multicopy homeobox gene, oocyte-specific homeobox 4 (*OBOX4*) [76].

*OBOX4* serves as a redundant gene to *Dux* and activates MERVL and MERVK elements in a *Dux*-independent manner. *OBOX4* can bind to the long terminal repeats of murine endogenous retroviruses with a leucine tRNA primer (such as MERVL) and murine endogenous retroviruses with lysine tRNA primer (such as MERVK), then affect the deposition of active epigenetic modifications (i.e. H3K4me3 and acetylation of histone 3 at lysine 27) in these regions, ultimately resulting in the activation of downstream ZGA genes [76]. Therefore, whether the spatial relationship between *OBOX4* and NPBs resembles that between *Dux* and NPBs deserves further in-depth study.

## 6. Totipotency can be controlled by regulating nucleolus precursor bodies

*Dux* is released in the presence of NBPs and the mature nucleolus has been found to be required for *Dux* loci silencing. Given that chromatin located close to the nucleolus has repressive histone modifications and low transcriptional levels [71,77,78], it was suggested that disruption of the mature nucleolus may enhance conversion to a totipotent state via detachment of *Dux* loci from the nucleolar periphery. Recent results have shown that short-term RNA Pol I inhibition or perturbation of nucleolar LLPS is sufficient to release *Dux* loci from the nucleolar periphery, thus converting ESCs into a two-cell-like state [9,10]. One study showed that CX-5461, a fluoroquinolone, blocks recruitment of the Pol I initiation factor SL1 to rDNA in ESCs and inhibits rRNA synthesis after 2 h of treatment, which induces morphological nucleolar remodelling that generates singular ring-like structures resembling embryo NPBs [9]. Following CX-5461 treatment and morphological nucleolar remodelling, *Dux* expression was significantly induced, then the expressions of two-cell-specific genes and MERVL transcripts were elevated and the proportion of two-cell-like cells within ESCs increased from less than 5% to 20%. In addition, as noted in the preceding text, the nucleolus is formed by LLPS of DNA, RNA and protein mixtures through weak hydrophobic interactions and 1,6-hexanediol (HDL), an aliphatic alcohol, can disrupt hydrophobic interactions. Following 1% HDL treatment for 2 h, phase separation was disrupted and *Dux* loci were released from the nucleolar periphery [9]. Essentially, the LLPS of the nucleolus and the formation of nucleolar periphery heterochromatin rely on nucleolar integrity. Once rRNA biogenesis is suppressed or the state of nucleolus LLPS is directly disrupted, the nucleolin/Kap1 complex will dissociate from nucleolar periphery heterochromatin, which in turn causes changes in the epigenetic state and reorganization of the three-dimensional structure of nucleolar periphery heterochromatin, including releasing *Dux* loci from the nucleolar periphery [9,10].

Differentiated somatic cells can be reprogrammed to totipotent embryos, which then form live cloned offspring, but cloning efficiency remains low [79–82]. In particular, transcriptional deregulation of *Dux* exists [83,84], resulting in endogenous retroviral silencing and severe ZGA defects in the vast majority of cloned embryos [16,18,19,85]. In theory, following SCNT, the reconstructed embryos need to undergo proper nuclear architecture reorganization (including nucleolar remodelling) to ensure the establishment of totipotency [86–88]. However, in early cloned embryos, somatic cell-like nucleoli frequently appear in pseudo-pronuclei, without the typical NPBs that appear in early fertilized embryos, and this may correspond to *Dux* silencing [20,21]. Moreover, cloned embryos exhibit multiple nucleoli maintenance in pseudo-pronuclei that is caused by an insufficient volume of the nucleolus, while in fertilized embryos, a few small nucleoli are fused into a single NPB [89]. According to the above phenomena, we hypothesized that after nuclear transfer, incomplete nuclear architecture remodelling, including abnormal NPBs in early cloned embryos, disturbs the full reprogramming; therefore, *Dux* fails to be reactivated, ultimately affecting ZGA and totipotency establishment. Recently, several methods targeting the nucleolus have been used to improve the development of cloned embryos. For instance, Liao *et al*. revealed that when cumulus cells treated with CX-5461 were used as donor cells for nuclear transfer, the deterioration of the mature nucleolus was accelerated in donor cells. In particular, the rate of blastocyst formation was elevated from 24% to 34% [90]. It is noteworthy that the volume of the nucleolus should reach a certain threshold so that the nucleolus performs its functions. For example, when the nucleolus volume in oocytes was reduced to half, 30% of embryos could still reach the blastocyst stage. Nevertheless, no embryo cleaved beyond the two-cell stage and no blastocyst formation occurred when less than half of the nucleolus remained in oocytes, this finding was also observed in embryos derived from completely enucleolated oocytes [91].

Likewise, Kyogoku *et al*. found that when extra NPBs were injected into enucleolated MII oocytes, the number of NPBs in one-cell stage cloned embryos decreased, with cleavage rate increasing significantly [89]. In the future, we believe that following more complete analysis of the components of NPBs, more experimental approaches will be found to regulate the functions of NPBs and contribute to the establishment of totipotency, ultimately improving the development of cloned embryos.

## 7. Conclusion

To date, several lines of evidence challenge the previous view that NPBs in zygotes only serve as a passive repository site for assembling fully functional nucleolus. In light of the indispensable roles played by NPBs during ZGA, we propose to describe the NPBs as a regulatory hub for chromatin organization that facilitates correct chromosome segregation and totipotency establishment. Along with advances in proteomic analysis, it is reasonable to believe that more components of NPBs will be identified, and the corresponding functions will also be dissected in future research. Moreover, more work is required to determine which genomic regions are associated with the periphery of NPBs and how the genome is organized around the NPBs. Recently, Peng *et al*. developed a nucleolus Hi-C experimental technique to enrich nucleolus-associated chromatin interactions, providing a method that may be helpful to identify high-confidence NADs and providing a global view of heterochromatin interactions organized around the nucleolus [92]. Such investigations will provide more insights into the mechanisms underlying the establishment of totipotency in early embryos, which, in turn, may provide new ideas for enhancing the reprogramming efficiency.

## Data Availability

This article has no additional data.
